# Long Term Follow-Up of Sarcopenia and Malnutrition after Hospitalization for COVID-19 in Conventional or Intensive Care Units

**DOI:** 10.3390/nu14040912

**Published:** 2022-02-21

**Authors:** Dan Levy, Margherita Giannini, Walid Oulehri, Marianne Riou, Christophe Marcot, Megane Pizzimenti, Lea Debrut, Anne Charloux, Bernard Geny, Alain Meyer

**Affiliations:** 1 Translational Medicine Federation of Strasbourg (FMTS), Team 3072 “Mitochondria, Oxidative Stress and Muscle Protection”, Faculty of Medicine, University of Strasbourg, 67000 Strasbourg, France; dan.levy@chru-strasbourg.fr (D.L.); margherita.giannini@chru-strasbourg.fr (M.G.); walid.oulehri@chru-strasbourg.fr (W.O.); marianne.riou@chru-strasbourg.fr (M.R.); megane.pizzimenti@hotmail.fr (M.P.); leadebrut@sfr.fr (L.D.); anne.charloux@chru-strasbourg.fr (A.C.); alain.meyer1@chru-strasbourg.fr (A.M.); 2 Physiology and Functional Exploration Service, University Hospital of Strasbourg, 67091 Strasbourg, France; 3 Department of Anesthesiology and Surgical Critical Care, University Hospital of Strasbourg, 67091 Strasbourg, France; 4 Department of Pneumology, University Hospital of Strasbourg, 67091 Strasbourg, France; christophe.marcot@chru-strasbourg.fr

**Keywords:** sarcopenia, COVID-19, muscle mass, muscle strength, malnutrition, densitometry, critical care, rehabilitation

## Abstract

Background: The post-COVID-19 condition, defined as COVID-19-related signs and symptoms lasting at least 2 months and persisting more than 3 months after infection, appears now as a public health issue in terms of frequency and quality of life alterations. Nevertheless, few data are available concerning long term evolution of malnutrition and sarcopenia, which deserve further attention. Method: Sarcopenia was investigated prospectively, together with weight evolution, at admission and at 3 and 6 months after hospital discharge in 139 COVID-19 patients, using the European Working Group on Sarcopenia in Older People (EWGSOP2) criteria, associating both decreased muscle strength and muscle mass, assessed, respectively, with hand dynamometer and dual-energy X-ray absorptiometry. Results: Of the 139 patients, 22 presented with sarcopenia at 3 months; intensive care units (ICU) length of stay was the sole factor associated with sarcopenia after multivariate analysis. Although the entire group did not demonstrate significant weight change, weight decreased significantly in the sarcopenia group (Five and eight patients, showing, respectively, >5 or >10% weight decrease). Interestingly, at 6 months, 16 of the 22 patients recovered from sarcopenia and their weight returned toward baseline values. Conclusions: Sarcopenia and malnutrition are frequently observed in patients hospitalized for COVID-19, even 3 months after infection occurrence, but can largely be reversed at 6 months after discharge. Enhanced patient care is needed in sarcopenic patients, particularly during long stays in an ICU.

## 1. Introduction

The severe acute respiratory syndrome coronavirus-2 (SARS-CoV-2) had spread worldwide by March 2020. Besides directly affecting COVID-19-infected patients, SARS-CoV-2 also had significant deleterious effects on subjects who had not been infected, often inducing malnutrition and reducing physical activity, especially in older persons [1,2].

Although many factors are associated with sarcopenia [3], COVID-19 infection, which ranges from asymptomatic forms to critical coronavirus-2019 (COVID-19) disease with high mortality rates [4], is recognized as an important cause of malnutrition and reduced muscle strength, even late after infection onset [5]. This is significant, since sarcopenia, defined by reduced muscle strength and mass, can result in handicap, poor quality of life, and increased mortality [6]. Sarcopenia in COVID-19 patients has been recently reported to be associated with increased complications and mortality [7,8]. Further, muscle mass and strength at admission appeared predictive of hospital length of stay, showing their implication in COVID-19 patients’ prognosis [9].

Such data are not surprising, since COVID-19 patients, as in other diseases, have a high risk of developing sarcopenia since they often present with hypoxemia, myalgia, elevated serum levels of creatine kinase and of pro-inflammatory cytokines (“cytokine storm”), including interleukin 6 (IL-6) and tumor necrosis factor alpha (TNF-α) which, similar to interferon, have been implicated in the pathogenesis of sarcopenia [10,11,12]. Finally, critical COVID-19 generally requires prolonged intensive care unit (ICU) hospitalization [13] that exposes these patients to ICU-acquired weakness [14]. Interestingly, Quaisar et al. [15], investigating subjects for sarcopenia diagnosis before the COVID-19 pandemic, reported that 26% of patients previously without sarcopenia demonstrated COVID-19 driven sarcopenia.

To date, at a time when long-term COVID-19 complication appears to be a public health issue, there are few data concerning the evolution of sarcopenia, determined by the gold standard densitometry method until 6-months after COVID-19 infection. We, therefore, determined, prospectively, sarcopenia occurrence and signs of malnutrition, together with clinical and cardiorespiratory functional characteristics in 139 patients three and six months after hospitalization for COVID-19 infection.

## 2. Methods

### 2.1. Population and Study Design

Consecutive patients, without exclusion criteria, who survived after hospitalization for COVID-19 but who had needed ICU and/or pulmonology department support at Strasbourg University Hospital between March 2020 and June 2020 were prospectively studied. According to the World Health Organization (WHO) guidelines [4], patients′ conditions were classified at the time of hospitalization as “mild”, “moderate”, “severe”, or “critical” disease. COVID-19 was diagnosed by positive real-time reverse-transcriptase polymerase chain reaction from a nasal and/or throat swab together with clinical signs, symptoms, and/or radiological findings suggestive of COVID-19 pneumonia [4]. Data regarding COVID-19-related hospitalization were collected by systematic medical chart review.

Written informed consent was obtained from all patients; the study was approved by the ethics committee of Strasbourg (RNI 2020—HUS N°7890) and the Institutional Review Board of the French Learned Society for Respiratory Medicine (CEPRO 2021—013).

### 2.2. Sarcopenia Determination

Sarcopenia was diagnosed using the Working Group on Sarcopenia in Older People (EWGSOP2) 2019 criteria [6]. Handgrip strength was assessed by a Jamar (Sammons Preston Rolyan, USA). Values under 27 kg for males and 16 kg for females were considered low (dynapenia). The appendicular skeletal muscle mass (ALM) was assessed by dual-energy X-ray absorptiometry (DXA Horizon W i, Hologic (Stephanix, France). ALM was adjusted for height (ALM/height^2^) and was considered pathological if <5.5 kg/m^2^ for women and <7.0 kg/m^2^ for men [6].

Severe sarcopenia was defined as a gait speed <0.8 m/s during a 4-m walking test and/or short physical performance battery score ≤8/12.

### 2.3. Nutritional Assessment

Nutritional status, as inferred from the weight and the body mass index (BMI: = body weight/height^2^), was obtained on admission, at 3 and at 6 months after discharge in the sarcopenia group. According to the French and Global Leadership Initiative on Malnutrition (GLIM) recommendations [16,17], “maltunitrion” was defined as the presence of (i) Etiologic Criteria: COVID-19 and (ii) phenotypic criteria: weight loss >5%. or BMI <20 (for patients <70 years old), or <22 (for patients ≥70 years old). Malnutrition was graded as “moderate” (weight loss ≤10% and BMI ≥18.5 for patients <70 years old, ≥20 for patients ≥70 years old) or “severe” (weight loss >10% and BMI <18.5 for patients <70 years old, <20 for patients ≥70 years old).

### 2.4. Other Parameters Investigated

Other evaluations included: anthropometric characteristics, comorbidities, hospitalization characteristics, pulmonary function tests and trans-thoracic echocardiography.

### 2.5. Statistical Analyses

Statistical analyses were performed using JMP software (version 7.0, SAS Institute Inc., Cary, NC, USA). Categorical variables were compared between groups using Fisher’s exact test or chi-square test. Quantitative variables were compared using t-test (normal distribution) or Mann–Whitney test (non-normal distribution). The normality of the distributions was verified with the Shapiro–Wilk test. Statistical significance was established at *p*-value < 0.05. Parameters significantly associated with sarcopenia were determined by applying multivariate analysis (logistic regression). To this aim, features displaying an association with sarcopenia 3 months after discharge with *p* < 0.05 at univariate analysis were included in the multivariate logistic regression analysis. Redundant features were not included in the multivariate analysis.

## 3. Results

**Figure 1:**
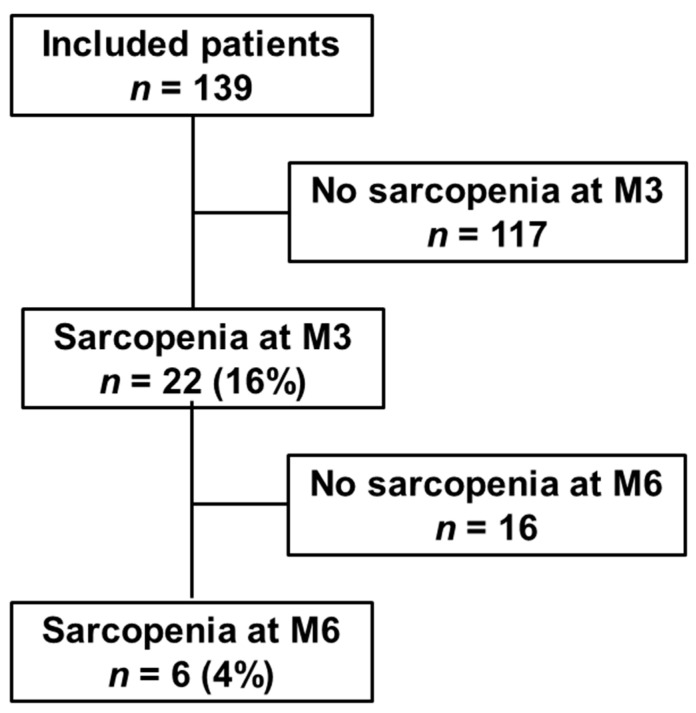
Flow-chart of the study. M3, three months after discharge following COVID-19-related hospitalization; M6, six months after discharge following COVID-19-related hospitalization.

One hundred and thirty-nine consecutive patients who survived after hospitalization for COVID-19 in the ICU and/or pulmonology department at Strasbourg University Hospital between March 2020 and June 2020 were prospectively included, as presented on Figure 1.

### 3.1. Patients Characteristics

As shown in Table 1, the 139 patients were mostly male (sex ratio 95/44), with a median age of 62 years (range 29 to 82). The majority of the patients had suffered from critical COVID-19 (*n* = 104, 75%), while the others had severe (*n* = 17, 12%) or moderate (*n* = 18, 13%) forms of the disease. On hospital admission, the patients were frequently obese (*n* = 60, 43%) and the majority had comorbidities (*n* = 97, 70%), mainly hypertension (*n* = 80, 58%) and type 2 diabetes mellitus (*n* = 44, 32%). There were no patients who presented with cancer or severe gastrointestinal disease. The majority (*n* = 99, 71%) had required ICU care. The median duration of ICU in the whole cohort was 7 days (range 0–115) and total hospital length of stay was 21 days (range 2–156).

### 3.2. Sarcopenia Evolution 3 and 6 months after Hospitalization for COVID-19

**Figure 2:**
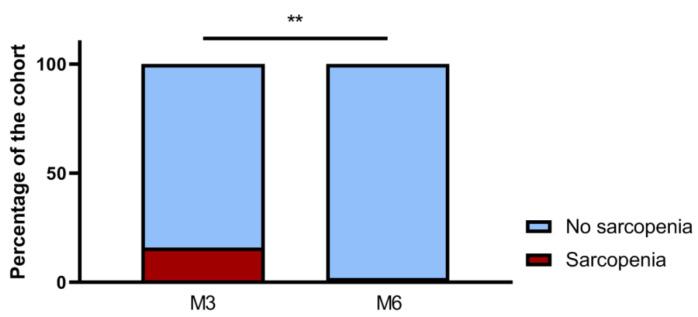
Prevalence of sarcopenia in the total cohort 3 months (M3) and 6 months (M6) after discharge following COVID-19-related hospitalization. M3, three months after discharge following COVID-19-related hospitalization; M6, six months after discharge following COVID-19-related hospitalization. **: *p* < 0.01.

As presented in Figure 2, three months after discharge following COVID-19-related hospitalization (M3), 22 patients (16%) were diagnosed with sarcopenia. Five patients (4%) showed severe sarcopenia. Comparison between sarcopenia and non-sarcopenia patients at M3 is shown in Table 1. All of the sarcopenia patients suffered from critical forms of COVID-19 as defined by the WHO (vs. 70% in the non-sarcopenia group, *p* = 0.01). Longer total hospital length of stay (41.5 days (6–156) vs. 20 days (2–120), *p* = 0.01), especially ICU length of stay (24 days (range 0–115) vs. 7 days (range 0–71), *p* = 0.01) and tracheostomy requirement (32% vs. 11%, *p* = 0.01) were significantly associated with sarcopenia 3 months after discharge. Patients with sarcopenia at M3 more frequently required rehabilitation after discharge (91% vs. 61%; *p* = 0.007) that lasted for 30 days (range 0–62). By contrast, no significant difference was found between the two groups regarding respiratory and cardiac functions at M3. With multivariate analysis, ICU length of stay was the sole variable associated with sarcopenia at M3 (*p* = 0.01).

Six months after discharge (M6), two thirds of the sarcopenia patients (*n* = 16/22) had recovered from sarcopenia, which persisted in 6 patients (4% of the cohort) and was severe in 3 of them (2% of the cohort). The median gain of strength was +10 kg (range −0.8; +24) and +15 kg (range +3; +19) on the right and left sides. The median gain of appendicular muscle mass was +0.52 kg/m^2^ (−0.42; +1.69). Comparison between patients with persistent sarcopenia and those who had recovered is shown in Table 2. Patients with persistent sarcopenia tended to be more frequently females (50% vs. 13%, *p* = 0.10), older (age 70 years vs. 63.5 years, *p* = 0.14) and had likely severe sarcopenia at M3 (60% vs. 25%, *p* = 0.28).

### 3.3. Weight Evolutions

When considering all patients, on admission, the weight was 87.2 kg (52–141); no significant change was observed 3 months later 86.1 kg (54–133).

**Figure 3:**
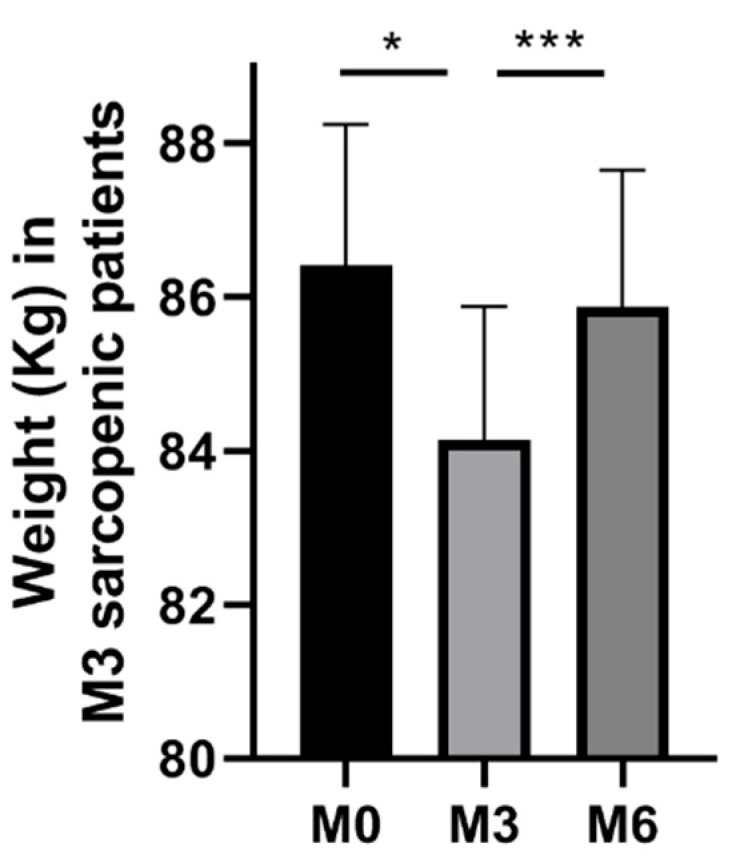
Weight at admission (M0), 3 months (M3) and 6 months (M6) after discharge following COVID-19-related hospitalization in the group of the patients with sarcopenia at M3. *: *p* < 0.05; ***: *p* < 0.001.

Nevertheless, in the subgroup of patients with sarcopenia, the weight significantly decreased at 3 months and was reversed by 6 months (Figure 3). More precisely, 5 and 8 patients showed, respectively, a weight decrease >5% or >10%.

## 4. Discussion

**Figure 4:**
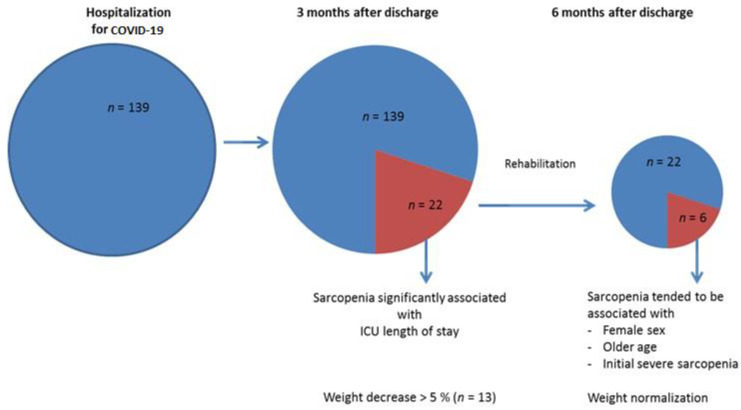
Sarcopenia and weight evolutions after hospitalization for COVID-19. ICU: intensive care unit.

The main results of this study are that 16 % and 4 % of the 139 patients hospitalized for COVID-19 demonstrated sarcopenia three and six months, respectively, after discharge. After a significant decrease, the sarcopenia patient’s weight returned toward baseline values six months after the hospitalization. ICU length of stay was the sole independent parameter associated with sarcopenia at three months. Patients with persistent sarcopenia at six months tended to be more frequently female, older and had severe sarcopenia at month three (Figure 4).

This study benefits from a prospective design and the recent definition of sarcopenia according to the EWGSOP2 consensus, taking into account both muscle mass and muscle strength, determined by adequate methods. To our knowledge, this is the first description of sarcopenia evolution—a significant nutritional challenge—until 6 months after hospitalization, mainly in the ICU, for COVID-19.

In a population with a majority of critical COVID-19 patients, we found a 16% prevalence of sarcopenia at 3 months after hospital discharge, which is consistent with previous data, although sarcopenia was not always, determined using gold standard methods. Several factors, including oxidative stress, inflammation, skeletal muscle mitochondrial impairments and impaired balance between muscle synthesis or degradation have been proposed to be responsible for sarcopenia occurrence in chronic diseases and in COVID-19 patients [10,11,12,18]. Here, ICU length of stay likely impairing muscle physiology through several pathways was the sole independent factor related to sarcopenia, confirming that sarcopenia is associated with complications in COVID-19 patients. Such results are also in line with a recent report showing that muscle strength and mass assessed, respectively, with handgrip and vastus lateralis cross sectional area have prognostic value in the case of COVID-19 infection [9]. In this view, we characterized patient’s evolution at 3 and 6 months after discharge, allowing us to determine sarcopenia evolution relatively late, which is important considering the ongoing burden of post-COVID-19 conditions. We decided to follow only sarcopenia patients at 6 months since the risk of worse outcome in non-sarcopenia patients was very low. Interestingly, only 4% of the 139 patients demonstrated sarcopenia six month after hospital discharge. This is encouraging and demonstrates that COVID-19-related sarcopenia can be reversed when appropriately diagnosed and treated. The degree of mobility was not systematically assessed during hospitalization in our study; however, reeducation should be performed as early as possible to limit both COVID- and reanimation-related deleterious effects on muscle strength and patients’ mobility [19]. In fact, most (91%) of the sarcopenia patients were rehabilitated, which likely explains their improvement in both muscle strength and mass.

Concerning the weight, when considering the entire group, no significant change was observed during the follow up. However, in the sarcopenia subgroup, 5 and 8 patients showed, respectively, moderate or severe malnutrition signs at 3 months. Accordingly, to previous data [8,20], Gerard et al. also recently reported that around 13% of the patients hospitalized for a severe form of COVID-19 were still severely malnourished 30 days after hospital discharge. The highest predictive factors of persistent malnutrition were ICU length of stay and male sex. At 6 months, the same authors reported that 43 out of the 53 patients previously impaired presented with persistent malnutrition, potentially related to obesity and to a greater initial weight loss [5]. In our study, a quasi-complete weight recovery was observed at 6 months in the sarcopenia group, thanks to the rehabilitation program. Indeed, similar to in other settings, multidisciplinary rehabilitation programs clearly improved the health status of COVID-19 patients [21]. We did not find clear predictors of long-lasting sarcopenia since comorbidities were similar between patients recovering from or with persistent sarcopenia. Nevertheless, older age and female sex, both associated with a lower response to rehabilitation in other settings, tended to be more frequent in patients with persistence of sarcopenia. Of note, an enhanced negative impact of COVID-19 on nutrition behavior has been reported in patients sharing the same characteristics [14,22,23].

Our study has some limitations. It was not possible to determine muscle mass and strength before COVID-19 infection and, thus, the prevalence of a possible pre-existent sarcopenia is unknown. Similarly, weight was not determined before hospitalization for COVID-19 and, thus, malnutrition severity might have been underestimated in some patients. Further, although interesting, nutritional intake was not systematically assessed. Nevertheless, in accordance with the standard of care, the French recommendations for hospitalized patients were applied [24]. In this view, early physical and nutritional assessment and education of COVID-19 patients should be integrated into the overall therapeutic strategy, which might open new therapeutic possibilities [25,26].

## 5. Conclusions

In conclusion, confirming that sarcopenia occurs after hospitalization for COVID-19, this prospective study shows that sarcopenia can be long-lasting in a subset of patients. Although sarcopenia generally responds well to rehabilitation, women and older patients may be at higher risk of persistent sarcopenia and, thus, deserve particular care and attention. In patients with serious COVID-19 infection, a specific follow-up should be proposed particularly in sarcopenia patients since, when present at 3 months, sarcopenia can persist in about one–third of cases at 6 months post discharge. As previously proposed, treatments should be holistic, and particularly focused on both nutritional support and personalized rehabilitation through exercise training [5].

## Figures and Tables

**Table 1:** Global distribution and comparison of the three months after discharge following COVID-19-related hospitalization (M3) features between sarcopenia and non-sarcopenia patients.

	Global Distribution (*n* = 139)	Sarcopenic Patients (*n* = 22)	Non-Sarcopenic Patients (*n* = 117)	Uni-Variate *p*-Value	Multi-Variate *p*-Value
Age, years (range)	62 (29–82)	65.5 (37–82)	62 (29–81)	0.07	—
Female, *n* (%)	44 (32)	5 (23)	39 (33)	0.45	—
Weigh, kg	86 (55–133)	79.5 (63–104)	89 (55–133)	0.008	—
Height, cm	172 (148–196)	170.5 (154–185)	172 (148–196)	0.71	—
BMI, kg/m^2^	29 (21–44)	26.6 (21.8–32)	29.4 (21–44)	0.004	—
Obesity, *n* (%)	60 (43)	5 (23)	55 (47)	0.03	—
COMORBIDITIES, *n* (%)	97 (70)	18 (82)	79 (68)	0.21	
Hypertension, *n* (%)	80 (58)	16 (73)	64 (55)	0.12	—
Diabetes, *n* (%)	44 (32)	9 (41)	35 (30)	0.31	—
Smoker, *n* (%)	28 (20)	6 (27)	22 (19)	0.36	—
Asthma, *n* (%)	4 (3)	1 (5)	3 (3)	0.50	—
COPD, *n* (%)	5 (4)	2 (9)	3 (3)	0.18	—
Asthma or COPD, *n* (%)	9 (6)	3 (14)	6 (5)	0.15	—
Chronic cardiac failure, *n* (%)	4 (3)	0	4 (3)	1	—
Chronic renal failure, *n* (%)	7 (5)	1 (5)	6 (5)	1	—
HOSPITALIZATION DATA					
COVID-19 severity, moderate/severe/critical, *n* (%)	18 (13)/17 (12)/104 (75)	0/0/22 (100)	18 (15)/17 (15)/82 (70)	0.01	0.89
Total hospital length of stay, day	21 (2–156)	41.5 (6–156)	20 (2–120)	0.01	—
ICU admission, *n* (%)	99 (71)	18 (82)	81 (69)	0.23	—
ICU length of stay, day (range)	7 (0–115)	24 (0–115)	7 (0–71)	0.01	0.01
Tracheostomy, *n* (%)	19 (14)	7 (32)	12 (10)	0.01	—
Rehabilitation after discharge, *n* (%)	93 (68)	20 (91)	67 (61)	0.007	0.16
Duration of rehabilitation, day (range)	14 (0–70)	30 (0–62)	0 (0–70)	0.002	—
MUSCLE EXPLORATIONS					
Dynapenia, *n* (%)	45 (33)	22 (100)	23 (20)	<0.001	—
Maximal right strength, kg(range)	28 (4–56)	18 (4–26)	30 (8–56)	<0.001	—
Maximal left strength, kg (range)	26 (3–56)	16.5 (3–25)	28 (3–56)	<0.001	—
Low muscle mass (DXA), *n* (%)	29 (22)	22 (100)	7 (6)	<0.001	—
Appendicular muscle mass, kg/m^2^ (range)	7.26 (4.24–13.3)	6.26 (4.92–6.98)	7.62 (4.24–13.3)	<0.001	—
Sarcopenia, *n* (%)	22 (16)				
Severe Sarcopenia, *n* (%)	5/139 (4)	5 (23)			
PULMONARY FUNCTION TESTS					
FEV, ml (range)	2705 (1120–4720)	2450 (1120–3940)	2710 (1350–4720)	0.75	—
FEV, %	97 (13–147)	96.5 (36–124)	97 (13–147)	0.68	—
FVC, ml (range)	3480 (1420–5430)	3470 (1420–5430)	3490 (1580–5290)	0.64	—
FVC, % (range)	100 (35–155)	99 (35–129)	101 (38–155)	0.44	—
FEV/FVC (range)	80 (40–100)	79 (57–91)	80 (40–100)	0.91	—
DLCO, % (range)	72 (21–115)	63 (40–88)	74 (21–115)	0.11	—
MMEF, % (range)	108 (41–175)	100 (46–175)	108 (41–175)	0.97	—
RV, % (range)	85 (37–148)	81 (48–135)	87 (37–148)	0.70	—
CARDIOLOGIC EXPLORATIONS					
LVEF, % (range)	62 (35–75)	62 (43–74)	62 (35–75)	0.48	—

BMI: bone mass index; ICU: intensive care unit; COPD: chronic obstructive pulmonary disease, DXA: dual X-ray absorptiometry; FEV: forced expiratory volume; FVC: forced vital capacity; DLCO: diffusion capacity for carbon monoxide; LVEF: left ventricular ejection fraction; MMEF: maximal mid-expiratory flow; RV: residual volume.

**Table 2:** Characteristics and evolution of the sarcopenic status at 6 months (recovery or not) in the subgroup of patients with sarcopenia three month after COVID-19 onset.

	Patients with Persistent Sarcopenia at M6 (*n* = 6)	Patients Recovering from Sarcopenia at M6 (*n* = 16)	*p*-Value
Age, years (range)	70.0 (64–80)	63.5 (37–82)	0.14
Female, *n* (%)	3 (50)	2 (13)	0.10
Weigh, kg	72.5 (70–82)	81 (63–104)	0.05
Height, cm	165 (154–180)	172 (160–185)	0.09
BMI, kg/m^2^	21.3 (23.5–30)	26.7 (21.8–32.0)	0.83
Obesity, *n* (%)	1 (17)	4 (25)	1.0
Comorbidities, *n* (%)	5 (83)	13 (81)	1.0
Hypertension, *n* (%)	5 (83)	11 (69)	0.63
Diabetes, *n* (%)	1 (17)	8 (50)	0.33
Smoker, *n* (%)	1 (17)	5 (31)	0.63
Asthma, *n* (%)	1 (17)	0	0.27
COPD, *n* (%)	0	2 (13)	1.0
Asthma or COPD, *n* (%)	1 (17)	2 (13)	1.0
Chronic cardiac failure, *n* (%)	0	0	1.0
Chronic renal failure, *n* (%)	1 (17)	0	0.27
HOSPITALIZATION DATA			
COVID-19 severity, moderate/severe/critical, *n* (%)	0/0/6	0/0/16	1.0
Total hospital length of stay, day (range)	48.5 (10–86)	39 (6–156)	0.48
ICU admission, *n* (%)	5 (83)	13 (81)	1.0
ICU length of stay, day (range)	38.5 (0–65)	22.5 (0–115)	0.55
Tracheotomy, *n* (%)	1 (17)	6 (38)	0.62
Rehabilitation after discharge, *n* (%)	5 (83)	15 (94)	0.48
Duration of rehabilitation, day (range)	43.5 (0–60)	26 (0–62)	0.67
MUSCLE EXPLORATIONS			
M3 Severe Sarcopenia, *n* (%)M6 Severe Sarcopenia, *n* (%)	3 (60)3 (60)	4 (25)0	0.281.0
Right strength variation between M3 and M6, kg	+5 (−0.8; +18)	+11 (4.5; +24)	0.30
Left strength variation between M3 and M6, kg	+10 (+3, +15)	+16.5 (+9; +19)	0.11
ALM variation between M3 and M6, kg/m^2^	+0.11 (−0.15; +0.89)	+0.63 (−0.42; +1.69)	0.16
PULMONARY FUNCTION TESTS			
FEV, ml (range)	2055 (1120–2250)	2820 (1220–3940)	0.03
FEV, %	72.8 (36–123)	97.5 (59.5–124)	0.25
FVC, ml (range)	2540 (1420–3360)	3666 (1580–5430)	0.07
FVC, % (range)	80.7 (35–121)	99 (62.5–128.6)	0.46
FEV/FVC (range)	81.5 (57–91)	79 (68–86)	0.61
DLCO, % (range)	60 (53-85.7)	64.5 (38–89)	0.67
MMEF, % (range)	83 (46–101)	109 (55–175)	0.14
RV, % (range)	102 (56–135)	79 (48–109)	0.33
CARDIOLOGIC EXPLORATIONS			
LVEF, % (range)	58 (43–74)	64.5 (51–74)	0.67

ALM: appendicular lean mass, BMI: bone mass index; ICU: intensive care unit; COPD: chronic obstructive pulmonary disease, M3, three months after discharge following COVID-19-related hospitalization; M6, six months after discharge following COVID-19-related hospitalization; FEV: forced expiratory volume; FVC: forced vital capacity; DLCO: diffusion capacity for carbon monoxide; LVEF: left ventricular ejection fraction; MMEF: maximal mid-expiratory flow; RV: residual volume.

## Data Availability

Data supporting reported results can be asked to the authors.
